# Coronary Microvascular Vasodilatory Function: Related Clinical Features and Differences According to the Different Coronary Arteries and Types of Coronary Spasm

**DOI:** 10.3390/jcm11010130

**Published:** 2021-12-27

**Authors:** Hiroki Teragawa, Chikage Oshita, Yuko Uchimura, Ryota Akazawa, Yuichi Orita

**Affiliations:** Department of Cardiovascular Medicine, JR Hiroshima Hospital, Hiroshima 732-0057, Japan; chikage-ooshita@jrhh.or.jp (C.O.); yuuko-uchimura@jrhh.or.jp (Y.U.); ryota-akazawa@jrhh.or.jp (R.A.); yuichi-orita@jrhh.or.jp (Y.O.)

**Keywords:** vasospastic angina, microvascular spasm, microvascular vasodilatory function

## Abstract

Background: In the clinical setting; the microvascular vasodilatory function test (MVFT) with a pressure wire has been used in ischaemia patients with non-obstructive coronary arteries (INOCA), including vasospastic angina (VSA) and microvascular angina (MVA). The exact factors that affect the microvascular vasodilatory function (MVF) in such patients are still unknown. We aimed to identify the factors, including clinical parameters and lesion characteristics, affecting the MVF in such patients. Methods: A total of 53 patients who underwent coronary angiography, spasm provocation tests (SPTs) and MVFTs were enrolled. In the MVFT, the coronary flow reserve (CFR) and index of microcirculatory resistance (IMR) were measured. Of the 53 patients, MVFT data in the left anterior descending coronary artery (LAD) were obtained from 49 patients, and the clinical parameters were checked in all of them. Based on the results of the SPT, coronary spasms were divided into focal spasm, diffuse spasm, and microvascular spasm (MVS). To assess the lesion characteristics influencing MVF, MVFT data were compared according to the types of coronary spasm and coronary vessels in 73 vessels of the 53 patients. Results: In 49 patients who underwent the MVFT in the LAD, the IMR was higher in active smokers (*n* = 7) than in former smokers (*n* = 15) and never smokers (*n* = 27, *p* < 0.01). In the 73 coronary arteries in this study, the type of coronary spasm did not correlate with the CFR or IMR, whereas a higher IMR were more frequently observed in cases of focal spasm than in cases of diffuse spasm (*p* = 0.03). In addition, the IMR was higher in the right coronary artery (RCA) than in the LAD (*p* = 0.02). Conclusion: These results indicate that the smoking status affected the MVF in patients with INOCA, suggesting the possibility of improvement in the MVF by smoking cessation in such patients. In addition, in the assessment of MVF, it may be important to take into account which coronary artery or types of coronary spasm are being evaluated.

## 1. Introduction

The assessment and treatment of epicardial coronary stenosis are well established [1]. However, in the clinical setting, many patients develop ischaemia with non-obstructive coronary arteries (INOCA) [2]. INOCA has several endotypes, such as vasospastic angina (VSA), microvascular spasm (MVS), microvascular vasodilatory dysfunction (MVD) and a combination of VSA and MVD [3,4,5]. INOCA is not always benign [4,6], and an effective treatment for it has not yet been determined. A recent expert consensus document has strengthened the importance of treatment according to the endotypes of INOCA [5]. Thus, the diagnosis of INOCA and the differentiation of its endotypes are more important than ever.

Non-invasive imaging techniques such as transthoracic Doppler echocardiography and positron emission tomography with coronary computed tomographic angiography can effectively diagnose INOCA [5]. However, these methods have some clinical limitations due to local expertise and availability or variability in assessing these modalities. Thus, an invasive method using guidewire-based coronary flow reserve (CFR) and/or microcirculatory resistance measurements is widely recommended [1] because an established protocol is followed, and the inter-observer variability is low.

In the clinical setting, it has been demonstrated that several factors, such as ageing, smoking, hypertension, dyslipidaemia and diabetes mellitus, are associated with MVD [5,7,8,9,10]. In any event, it would be beneficial to elucidate the clinical indicators associated with these MVDs so that we could intervene in their treatment. Other clinical questions are whether or not microvascular vasodilatory function (MVF) varies with the type of coronary spasm or with each coronary artery. Recently, it has been reported that the prognosis in patients with VSA and MVD is worse than those without MVD [4]. Thus, it is also clinically important to elucidate the relationship between the type of coronary spasm and MVF. Furthermore, MVF testing (MVFT) has been often measured in the left anterior descending coronary artery (LAD) [4,11], but it is not known whether it varies by coronary artery. This study aimed to investigate the relationship between the MVF and clinical parameters and whether the function varies according to the lesion characteristics of the coronary artery, including the types of coronary spasm.

## 2. Materials and Methods

### 2.1. Study Population

**Figure 1:**
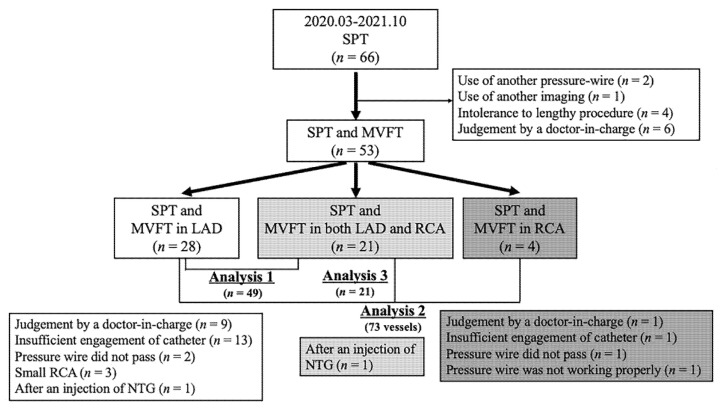
This is a figure of the study flowchart. Analysis 1 examined the relationship between the clinical parameters and IMR in 49 patients in whom MVFT data could be obtained in the LAD. Analysis 2 examined the relationship between MVFT data and lesion characteristics in 73 coronary vessels. Analysis 3 compared MVFT data between LAD and RCA in 21 patients in whom MVFT data could be obtained in both the LAD and RCA. LAD, left anterior descending coronary artery; MVFT, microvascular vasodilatory function test; NTG, nitroglycerin; RCA, right coronary artery; SPT, spasm provocation test.

This was a retrospective study of 66 patients with chest pain on whom we performed coronary angiography (CAG) and a spasm provocation test (SPT) between March 2020 and October 2021 at our institution (Figure 1). In two patients with moderate tandem lesions, another pressure guidewire was used. In one patient with VSA, coronary angioscopy was performed. MVFT was not performed on 10 patients because of either their intolerance to lengthy procedures (*n* = 4) or the judgement of the doctor-in-charge (*n* = 6). Thus, 53 patients who underwent both SPT and MVFT with a pressure wire were enrolled. The patient selection process and the number of analysed patients who underwent each procedure are presented in Figure 1. We excluded patients who had moderate coronary stenosis (% stenosis ≥ 30%) or moderate chronic kidney disease (CKD) with an estimated glomerular filtration rate (eGFR) of <45 mL/min/1.73 m^2^ or a history of heart failure or percutaneous coronary interventions. The ethics committee of JR Hiroshima Hospital approved this study (2021-37). Informed consent was obtained from all patients.

### 2.2. Coronary Function Test (CFT)

The methods used for the SPT at our institution have been previously described [12]. In brief, an SPT is performed after a standard diagnostic CAG employing the percutaneous brachial approach using a 5-Fr sheath diagnostic Judkins-type catheter. After the initial CAG, 50, 100 and 200 μg of acetylcholine (ACh) were infused into the left coronary artery (LCA) for 20 s at 3-min intervals [13]. CAG was performed immediately after coronary spasms were induced or the maximum ACh infusion was completed. If a coronary spasm was induced but improved spontaneously, a right coronary artery (RCA) SPT was then performed without the intracoronary injection of nitroglycerin (NTG) into the LCA. Once the SPT for the RCA was finished, CAG was repeated after an NTG injection into the LCA. If a prolonged coronary spasm was provoked by ACh infusion into the LCA or it induced haemodynamic instability, an intracoronary injection of 0.3 mg of NTG was administered. After spasm provocation in the LCA, 20, 50 and 80 μg of ACh were infused into the RCA for 20 s at a 3-min interval. CAG was performed immediately after coronary spasms were induced or the maximum ACh infusion was completed. After an intracoronary injection of 0.3 mg of NTG, the final CAG of the RCA was performed. We could not determine (NA) when the subsequent SPT was negative after an inevitable use of NTG.

The methods employed for the MVFT were as described in previous papers [11,14]. A pressure–temperature sensor-tipped PressureWire X Cabled Guidewire (Abbot Laboratories, Abbot Park, IL, USA) was used. Parameters were assessed using the CoroFlow software program (Coroventis, Uppsala, Sweden). The PressureWire was safely advanced in the LAD and RCA distally. To derive the resting mean transit time (Tmn), a thermodilution curve was obtained with three injections of 3-mL saline at room temperature. Hyperaemia was induced by intravenous infusion of adenosine triphosphate (160 µg/kg/min) through the peripheral vein. The hyperaemic proximal aortic pressure (Pa), distal arterial pressure (Pd) and hyperaemic Tmn were measured during maximal hyperaemia. The fractional flow reserve (FFR) was calculated as the lowest average of three consecutive beats during stable hyperaemia. CFR was calculated using the formula resting T_mn_/hyperaemic T_mn_. The index of microcirculatory resistance (IMR) was calculated using the formula Pd × T_mn_ during hyperaemia. To avoid the occurrence of pressure drift in the measurement of these parameters, we routinely calibrated the aortic pressure in the catheter and the pressure obtained by the PressureWire before measuring these parameters in each coronary artery. In addition, we confirmed that there was no pressure drift between the pressure obtained from the withdrawal of the PressureWire and the aortic pressure.

### 2.3. Definitions of CFT

The method used for measuring the diameter of the coronary artery has been described previously [12]. We selected spastic and atherosclerotic segments for quantitative analysis. The average value of three measurements was used for analysis. Changes in the coronary artery diameter in response to the ACh and NTG infusions were expressed as percentage changes from baseline angiographic measurements. Lesions with >20% stenosis were defined as atherosclerotic lesions. As previously reported [15,16], we investigated whether a myocardial bridge, defined as the systolic narrowing of the coronary artery diameter by >20% compared with that in diastole, was present. We also checked the frequency of a dominant RCA (an RCA with both the posterior descending artery and the posterolateral branch) [17].

Coronary spasm was defined as >90% narrowing of the epicardial coronary arteries on angiography during SPT, the presence of characteristic chest pain and/or ST-segment deviation identified via electrocardiography (ECG) [18,19]. A focal spasm was defined as a transient vessel narrowing of >90% within the borders of one isolated coronary segment as defined by the American Heart Association [20]. A diffuse spasm was defined as a 90% diffuse vasoconstriction observed in ≥2 adjacent coronary segments of the coronary arteries [21]. MVS was defined as the absence of angiographic coronary spasm accompanied by characteristic chest pain and ST-T ECG changes during SPT [5,22]. MVD was defined as the presence of IMR values of ≥25 units or CFR values of <2.0 [1,5].

### 2.4. Definitions of Clinical Parameters

We classified the patients according to smoking status as active smokers, former smokers (had stopped smoking for at least 1 month) or never smokers. Hypertension was defined as a systolic blood pressure of ≥140 mmHg, a diastolic blood pressure of ≥90 mmHg or the use of antihypertensive medication. We measured the levels of triglycerides, low-density lipoprotein cholesterol, fasting blood glucose, haemoglobin A1C, creatinine, C-reactive protein and N-terminal pro-brain natriuretic peptide. The eGFR (mL/min/1.73 m^2^) was calculated using the standard formula, and the presence of CKD was defined using standard criteria [23]. Dyslipidaemia was defined as a low-density lipoprotein cholesterol level of ≥120 mg/dL or the use of medications for dyslipidaemia. Diabetes mellitus was defined as a fasting blood sugar level of ≥126 mg/dL, haemoglobin A1C level of ≥6.5% or use of anti-diabetic medications. Metabolic syndrome (MtS) was also defined using standard criteria [24]. The left ventricular ejection fraction (LVEF) was measured via echocardiography. The left ventricular mass index (LVMI) was calculated using the formula of Devereux and Reichek [25,26]. As demonstrated previously [27], the flow-mediated dilation (FMD) and NTG-mediated dilation (NMD) of the brachial artery, which are objective measures of the endothelium-dependent and endothelium-nondependent functions, respectively, were evaluated using the UNEXEF device (UNEX Corp, Nagoya, Japan). Finally, peripheral endothelial function was measured via reactive hyperaemia peripheral artery tonometry (RH-PAT) using the Endo-PAT2000 device (Itamar Medical, Caesarea, Israel). The reactive hyperaemia index (RHI) was calculated as demonstrated previously [28].

### 2.5. Statistical Analyses

Continuous data are expressed as median values with interquartile ranges. The relationship between the IMR and clinical parameters was assessed using the Wilcoxon signed-rank test or Spearman’s rank correlation coefficient. Multiple comparisons in nonparametric methods were used to compare the IMR between the groups for smoking. The relationship between MVFT data and lesion characteristics was evaluated using the Wilcoxon signed-rank test or χ^2^ analysis. Logistic regression analysis was employed to determine the presence of MVD. In the 21 patients with MVFT data from both LAD and RCA, data were displayed using the Bland–Altman plots and data comparisons were performed using the Wilcoxon signed-rank test. All statistical analyses were conducted using JMP Ver. 16 (SAS Institute Inc., Cary, NC, USA). A *p* value of <0.05 was considered significant.

## 3. Results

A total of 53 patients (median age, 69 years; 24 men and 29 women) underwent CAG, SPT and MVFT. Of them, 21 experienced an MVFT in both the LAD and the RCA, 28 underwent an MVFT only in the LAD and 4 patients underwent an MVFT only in the RCA (Figure 1). The reasons for the non-performance of the MVFT in both coronary arteries were as follows: judgement by the treating physician (*n* = 10 vessels), insufficient engagement of the catheter during the insertion of the pressure wire or injection of saline (*n* = 1 vessel, LAD; *n* = 13 vessels, RCA), difficulty in inserting the pressure wire into the distal coronary artery (*n* = 3 vessels), a small RCA (*n* = 3 vessels, SPT was also not performed), and NTG administration during coronary spasm in another coronary artery (*n* = 2 vessels). Thus, subsequent analyses of the relationship between the clinical parameters and IMR were conducted on 49 patients in whom MVFT data could be obtained in the LAD (Analysis 1). The relationship between MVFT data and lesion characteristics was determined in 73 coronary vessels (Analysis 2). Finally, comparisons of MVFT data between LAD and RCA of the same patient were performed in 21 patients in whom MVFT data could be obtained in both the LAD and RCA (Analysis 3).

### 3.1. Relationship between Patients’ Characteristics and MVFT Data (Analysis 1)

**Figure 2:**
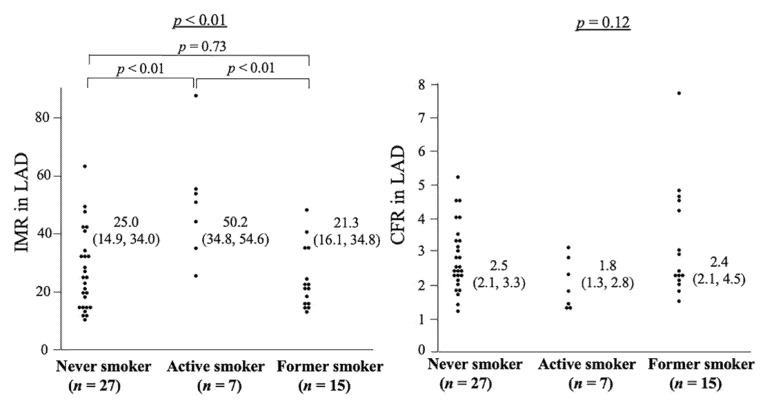
This is a figure of IMR and CFR regarding the smoking status in 49 patients who had MVFT in LAD (Analysis 1). The **left** panel shows the relationship between smoking status and IMR: active smokers had significantly higher IMR than never smokers and former smokers. The **right** panel shows the association between smoking status and CFR, which is not significant among the three groups. CFR, coronary flow reserve; IMR, index of microcirculatory resistance, LAD, left anterior descending coronary artery; MVFT, microvascular vasodilatory function test.

The characteristics of 49 patients in whom MVFT data could be obtained in the LAD are presented in Table 1. The factors shown in Table 1, except for smoking status, were not associated with either the CFR or the IMR. The values of LVMI, FMD, RHI and presences of a myocardial bridge or VSA did not affect the CFR and IMR. The CFR correlated negatively with the IMR (*p* < 0.01). With regard to the smoking status, the IMR values were 50.2 (34.8, 54.6), 21.3 (16.1, 34.8) and 25.0 (14.9, 34.0) in active smokers (*n* = 7), former smokers (*n* = 15) and never smokers (*n* = 27, *p* < 0.01), respectively, whereas those that were not associated with the CFR were 1.8 (1.3, 2.8) in active smokers, 2.4 (2.1, 4.5) in former smokers and 2.5 (2.1, 3.3) in never smokers (*p* = 0.12, Figure 2).

### 3.2. Relationship between MVFT Data and Lesion Characteristics (Analysis 2)

In 28, 21 and 4 patients had MVFT data in the LAD, in both the LAD and the RCA and, in the RCA, respectively; thus, a total of 73 sets of MVFT data for all coronary vessels were analysed (Table 2). Atherosclerosis (% stenosis < 30%) did not affect the CFR or IMR, while it reduced the baseline Pd/Pa (*p* = 0.04) and FFR (*p* = 0.01). In the vessel analyses, the baseline Pd/Pa, FFR and IMR in the RCA (*n* = 24) were significantly higher than those in the LAD (*n* = 49). The presence of MVD was higher in the RCA than in the LAD (*p* = 0.01). The CFR value did not significantly differ between the LAD and RCA. The types of coronary spasm did not significantly differ between the RCA and LAD (*p* = 0.07). Regarding the types of spasm, FFR values was different in the 4 groups (*p* = 0.03), however, the baseline Pd/Pa, CFR and IMR were not different in the 4 groups. The frequency of MVD was different in the four groups (*p* = 0.046). In the comparisons of MVFT data between focal and diffuse spasms, the FFR (*p* = 0.03), IMR (*p* = 0.03) and presence of MVD (*p* < 0.01) were higher in focal spasms than in diffuse spasms. Logistic regression analysis revealed that focal spasm and the measurement in the RCA were factors associated with MVD (Table 3).

### 3.3. Relationship between MVFT Data in the LAD and RCA (Analysis 3)

**Figure 3:**
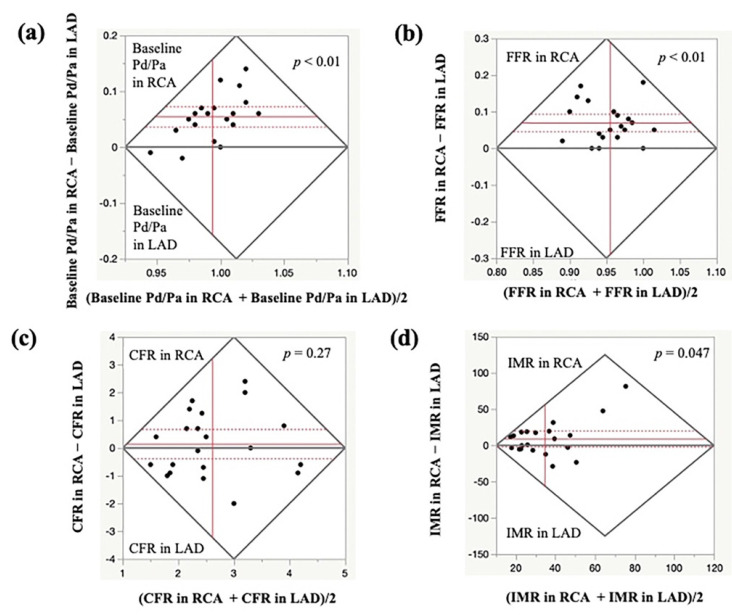
Comparison of MVFT data in the LAD and RCA. The Bland–Altman plots show the baseline Pd/Pa (**a**), FFR (**b**), CFR (**c**) and IMR (**d**) in the LAD and RCA. The baseline Pd/Pa, FFR and IMR were significantly higher in the RCA than in the LAD, whereas no significant difference was observed in the CFR values between the LAD and RCA. CFR, coronary flow reserve; FFR, fractional flow reserve; IMR, index of microcirculatory resistance; LAD, left anterior descending coronary artery; MVFT, microvascular vasodilatory function test; RCA, right coronary artery.

**Figure 4:**
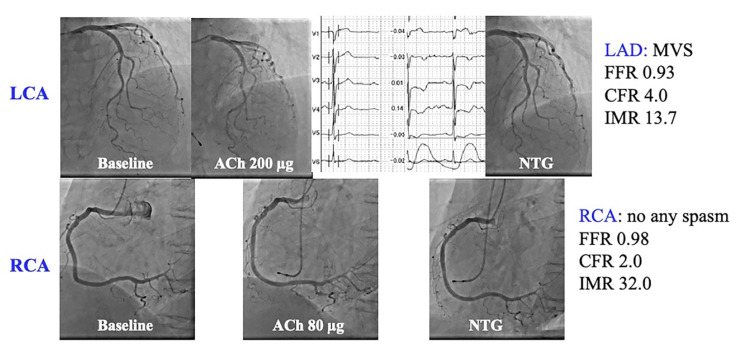
A representative case (Case 11 in Table 4).

The data of 21 patients in whom SPT and MVFT were performed in both LAD and RCA are presented in Table 4 and Figure 3. The baseline Pd/Pa, FFR and IMR values in the RCA were significantly higher than those in the LAD (*p* < 0.01, *p* < 0.01 and *p* < 0.05, respectively), whereas the CFR values did not significantly differ between the LAD and RCA (*p* = 0.27). A higher IMR in the RCA than in the LAD was detected in 12 out of 21 patients (57%). A dominant RCA was detected in 17 out of 21 patients (81%). No significant relationship was observed between a higher IMR in the RCA than in the LAD and a dominant RCA (*p* = 0.75). The types of spasm types between LAD and RCA matched in only 7 of 21 cases (33%). MVD was detected in 14 out of 21 LAD (67%) and 17 out of 21 RCA (81%) and there was a coincidence in 14 out of 21 patients (67%). Out of seven patients with disparities in the presence of MVD between the LAD and the RCA, the types of spasm in the LAD and the RCA were different in 5 out of 7 patients (71%). Figure 4 presents a representative case (Case 11) with normal MVF in the LAD with MVS (CFR, 4.0; IMR, 13.7) and MVD in the RCA without any types of spasm (CFR, 2.0; IMR, 32).

A 79-year-old woman complained of chest pain at rest. LAD SPT revealed no inductions of epicardial coronary spasm (upper panels). With chest pain and precordial inverted T waves during SPT in the LAD, MVS was considered in the LAD. Contrarily, the SPT for the RCA showed no inductions of angiographic and electrocardiographic changes. The MVFT revealed normal CFR and IMR in the LAD with MVS. There was reduced CFR and increased IMR in the RCA without any types of spasm. This patient might have different MVFs in the RCA and LAD.

CFR, coronary flow reserve; IMR, index of microcirculatory resistance; MVF, microvascular vasodilatory function; MVFT, microvascular vasodilatory function test; MVS, microvascular spasm; SPT, spasm provocation test.

## 4. Discussion

In this study, we investigated MVF in patients who underwent SPT and MCFT to evaluate their chest symptoms. We found that (1) smoking status, especially active smoking may increase IMR; (2) the focal spasm type and RCA may affect IMR and (3) the baseline Pd/Pa, FFR and IMR in the RCA were higher than those in the LAD, although the CFR did not significantly differ between the two vessels.

Factors such as ageing, smoking, hypertension, dyslipidaemia and diabetes mellitus are associated with MVD [5,7,8,9,10]. In this study, smoking status, especially active smoking, was associated with an increased IMR, which is in agreement with the results of other studies [7,9]. However, other factors were not associated with the MVFT data. In general, smoking has been known to increase oxidative stress, leading to vascular inflammation, impaired prostacyclin production, and vascular dysfunction [29,30]. Moreover, 60% of our patients had VSA, and smoking is one of the major risk factors for VSA [31]. This population and the small sample size may have contributed to the slight difference in the results. In addition, since this study was cross-sectional, it is unclear from the results whether smoking cessation improves MVD. However, considering the fact that there was no significant difference in the MVFT data between former smokers and never smokers, smoking cessation may be a valuable treatment for MVD. 

The present study demonstrated that the IMR value in the RCA was higher than that in the LAD, which is in agreement with the results reported by Murai et al. [10]. They speculated that the spatial heterogeneity of myocardial flow in different vascular beds caused the IMR to be higher in the RCA. The metabolic difference between the left and right ventricles may cause a difference in the myocardial flow between the LAD and the RCA. In the present study, we checked the presence of a dominant RCA, which could lead to more spatial heterogeneity of myocardial blood flow in the RCA. We could not demonstrate such a relationship, neither can we draw a definite conclusion owing to the small number of study participants. On the other hand, our results indicate that the distal pressures of the RCA at rest and during hyperaemia, indicated by baseline Pd/Pa and FFR, were significantly higher than those of the LAD. These findings are due to the lower anatomical position of the pressure wire tip in the distal RCA than in the distal LAD [32,33,34]. Furthermore, our results reveal no statistically significant difference between the CFR values in the LAD and RCA. This suggests that the increased IMR in the RCA may often be caused by the differences in the distal LAD and RCA pressures used in the formula for calculating IMR rather than by a real difference in the MVF. Other methods for assessing the MVF using a Doppler flow guidewire may be needed to confirm our results. Finally, methodological issues may also be the cause. Although we thoroughly checked for calibration before measuring each coronary artery and for drift after measurement, we still could not deny the possibility that a systematic error occurred. In summary, the difference in the IMR between the LAD and the RCA may be due to (1) the spatial heterogeneity in myocardial blood flow between the LAD and the RCA due to the differences in the perfused myocardial territory and/or metabolism in the left and right ventricles, (2) the anatomical differences in the distal pressure and (3) other factors, such as a systematic error. However, in our representative case (Figure 4), the CFR also significantly differs between the LAD and the RCA, indicating the presence of diverse MVFs. In such cases, calcium-channel blockers seem to be the first choice because of the presence of MVS; however, to evaluate the drug efficacy and prognosis in such cases, careful observation and data collection, such as from a multicentre registry, will be needed in the future.

Regarding the relationship between MVF and VSA, the presence of MVD has been noted in patients with VSA [4,35,36], partially owing to the increased coronary perivascular adipose tissue in VSA patients [37]. In this study, we could not find any relationship in terms of CFR and IMR between VSA and non-VSA patients, partly because of the difference in patients’ characteristics and a small number of studied patients. However, we demonstrated the relationship between focal spasm and MVD. With regard to the types of spasm and CFR, a previous study revealed that the CFR was reduced in diffuse spasm [38]. Although there are differences in the drugs used to induce coronary spasms, the definitions of diffuse spasms and the methods for assessing the CFR may influence the results. It has been demonstrated that VSA patients with focal spasms had a poorer prognosis than those with diffuse spasm [21]. It was also demonstrated that VSA patients with MVD had a poorer prognosis than those without [4]. The results of these studies may suggest the close link between focal spasm and MVD. Another possible explanation was the insufficient microvascular vasodilation due to the standard dose of NTG, especially in focal spasm. Suda et al. demonstrated that the intracoronary infusion of fasudil, a Rho-kinase inhibitor, improved the IMR [4], and these data could support the fact that the IMR was increased due to inadequate microvascular vasodilatation in the case of focal spasm. In the case of focal spasm, it might be better to use a more sufficient dose of NTG or other coronary dilators that dilate the microvascular blood vessels and take a little time to assess the MVF. Finally, some attention has been focused on the MVF in patients with MVS [4,35], showing that MVD is not always present in MVS. Our results also did not indicate a significantly higher frequency of MVD in MVS. However, the number of analysed patients was insufficient. Thus, further studies with a larger sample size may be needed to confirm our results.

Our results show that MVF, especially IMR, may vary from vessel to vessel, depending on coronary artery anatomy, or on the function of each coronary artery itself. Although it takes time to perform CFT, it may suggest that it is more important to evaluate each coronary artery rather than on a patient-by-patient basis. Again, owing to the small number of cases, it reiterates the fact that large multicentre registries and prospective consecutive case studies are needed.

This study has some limitations. First, the sample size of present study was relatively small, especially in the subgroup analyses, such as the active smokers (*n* = 7). This may have introduced some type II errors. Second, this was a retrospective study conducted in one institution, with a non-consecutive design. Thus, further prospective studies with more participants or a multicentre registry study will be needed to confirm our results. Third, routine CFT was performed using a 5-Fr catheter, and in some patients, it was not possible to engage the catheter in the ostium of the coronary artery, especially the RCA. Since we excluded patients, whose data were not completely available, the results obtained from the RCA may have varied, leading to the difference in coronary MVF between the RCA and the LAD. Further studies using a 6-Fr guiding catheter will be needed to further investigate our results. Fourth, the median IMR in this study was approximately 25, which was overall higher than those from the usual INOCA studies [4]. The frequency of hypertension, time off coronary dilation drugs, blood pressure during the test, and NTG and ATP load were considered as possible causes, but nothing definite was found; there may be an effect of RCA vessels and frequency of focal spasm, but the exact mechanism was not elucidated. Finally, we performed MVFT in the LAD and RCA but not in the left circumflex coronary artery (LCX) as we had no information on the MVF in the LCX.

## 5. Conclusions

Our results indicated that smoking status might be associated with an increased IMR, although there have been no clinically confident markers suggestive of MVD. The results suggest that smoking cessation might be one of the possible treatments for such MVD in our patients. The results also indicate that the IMR may be elevated during RCA and in focal spasm. It may be important to carefully examine the vessel that is being measured and to take measures such as adding sufficient coronary dilator and taking some time before measuring the IMR in case of focal spasm. However, the sample size of this study was small, and the investigation needs to be repeated with a larger sample size.

## Figures and Tables

**Table 1:** Patients’ characteristics of 49 patients who had MVFT in LAD.

Factors	Numbers or Values	Relationship between Factors and CFR, *p* Value	Relationship between Factors and IMR, *p* Value
Age (years)	69 (53, 76)	0.54	0.65
Men/Women	22/27	0.16	0.54
Body mass index	23.5 (21.8, 25.7)	0.13	0.37
Coronary risk factors			
Smoker (active/former/never)	7/15/27	0.12	<0.01
Hypertension	28 (57%)	0.65	0.15
Dyslipidaemia	26 (53%)	0.94	0.35
Diabetes mellitus	6 (13%)	0.92	0.36
Presence of MtS	7 (14%)	0.08	0.39
Presence of CKD	9 (18%)	1.00	0.53
Blood chemical data			
LDL-cholesterol (mg/dL)	100 (88, 123)	0.71	0.16
Triglyceride (mg/dL)	99 (82, 156)	0.24	0.77
Fasting blood sugar (mg/dL)	99 (90, 109)	0.32	0.43
Haemoglobin A1C (%)	5.9 (5.6, 6.2)	0.42	0.56
CRP (mg/dL)	0.05 (0.03, 0.11)	0.78	0.99
eGFR (mL/min/1.73 m^2^)	67.0 (61.4, 74.7)	0.73	0.25
NT-proBNP (pg/mL)	92 (45, 195)	0.17	0.12
Echocardiography			
LVEF (%)	66 (62, 70)	0.22	0.86
LVMI (g/m^2^)	80 (68, 94)	0.48	0.29
Peripheral endothelial function			
FMD (%)	3.5 (2.3, 5.3)	0.55	0.82
NMD (%)	15.8 (10.3, 18.1)	0.14	0.16
RHI	1.57 (1.43, 2.10)	0.71	0.69
CAG SPT MVFT			
Myocardial bridge	13 (27%)	0.18	0.28
VSA	31 (63%)	0.58	0.33
MVS	9 (18%)	0.43	0.35
Baseline Pd/Pa in LAD	0.96 (0.95, 0.98)	0.84	0.65
FFR in LAD	0.92 (0.89, 0.94)	0.94	0.84
CFR in LAD	2.4 (2.0, 3.3)	(−)	<0.01
IMR in LAD	25.0 (16.1, 40.3)	<0.01	(−)

Numbers were expressed as the numbers (percentage) and values were expressed as the median with interquartile ranges. CAG, coronary angiography; CFR, coronary flow reserve; CKD, chronic kidney disease; CRP, C-reactive protein; eGFR, estimated glomerular filtration rate; FFR, fractional flow reserve; FMD, flow-mediated dilation; IMR, index of microcirculatory resistance; LAD, left anterior descending coronary artery; LDL, low-density lipoprotein; LVEF, left ventricular ejection fraction; LVMI, left ventricular mass index; MtS, metabolic syndrome; MVFT, microvascular vasodilatory function test; MVS, microvascular spasm; NMD, nitroglycerin-mediated dilation; NT-proBNP, N-terminal pro-brain natriuretic peptide; RHI, index of reactive hyperemia; SPT, spasm provocation test; VSA, vasospastic angina. (−) means that the test was not performed because they were for the same indexes.

**Table 2:** Relationship between MVFT and lesion characteristics.

Lesion Characteristics		No.	Baseline Pd/Pa	*p* Value	FFR	*p* Value	CFR	*p* Value	IMR	*p* Value	*MVD*	*p* Value
Atherosclerosis	(+)	26	0.97 (0.95, 0.98)	0.04	0.92 (0.85, 0.96)	0.01	2.4 (1.9, 3.5)	0.88	25.1 (20.9, 41.9)	0.72	15 (57%)	0.14
	(−)	47	0.98 (0.96, 1.00)		0.94 (0.92, 0.99)		2.5 (2.0, 3.3)		28.8 (19.5, 41.2)		35 (74%)	
Vessels	LAD	49	0.96 (0.95, 0.98)	<0.01	0.92 (0.89, 0.94)	<0.01	2.4 (2.0, 3.3)	0.83	25.0 (16.1, 40.3)	0.01	29 (59%)	0.01
	RCA	24	1.02 (1.00, 1.03)		1.00 (0.96, 1.02)		2.7 (1.8, 3.3)		36.6 (25.3, 46.1)		21 (88%)	
Types of spasm	Focal spasm	24	0.97 (0.95, 1.00)	0.15	0.94 * (0.91, 0.99)	0.03	2.4 (1.8, 3.2)	0.09	33.4 * (25.1, 48.4)	0.15	21 * (88%)	0.05
	Diffuse spasm	15	0.96 (0.94, 0.98)		0.92 (0.87, 0.94)		2.8 (2.1, 4.5)		23.0 (16.1, 35.0)		7 (47%)	
	MVS	12	0.97 (0.95, 1.00)		0.94 (0.90, 0.97)		2.3 (1.7, 2.6)		31.6 (13.8, 40.1)		8 (67%)	
	None	22	0.98 (0.95, 1.02)		0.96 (0.92, 1.01)		2.9 (2.3, 3.9)		25.3 (21.8, 44.2)		14 (64%)	

Numbers were expressed as the numbers (percentage) and values were expressed as the median with interquartile ranges. CFR, coronary flow reserve; FFR, fractional flow reserve; IMR, index of microcirculatory resistance; LAD, left anterior descending coronary artery; MVFT, microvascular vasodilatory function test; MVS, microvascular spasm; Pa, aortic pressure; Pd, distal pressure; RCA, right coronary artery. * *p* < 0.05 vs. diffuse spasm.

**Table 3:** Logistic regression analyses of lesion characteristics of the presence of MVD.

Factors	Estimate	95% CI	χ^2^	*p* Value
Atherosclerosis	−0.36	−0.99–0.25	1.34	0.25
Vessels				
RCA	0.90	0.20–1.75	5.37	0.02
Types of spasm				
Focal spasm	1.41	0.41–2.63	6.49	0.01
Diffuse spasm	−0.77	−1.77–0.20	2.40	0.12
MVS	−0.04	−1.16–1.13	0.01	0.93
				R^2^ = 0.19

CI, confidence interval; MVD, microvascular vasodilatory dysfunction; MVS, microvascular spasm; RCA, right coronary artery.

**Table 4:** Data in patients who performed MVFT in both LAD and RCA.

**Case No.**	**Age** **(Years)**	**Gender**	Diagnosis	LAD								RCA								IMR in RCA> in LAD	Dominant RCA
				Atherosclerosis	Epi- Spasm	Type of Spasm	Baseline Pd/Pa	FFR	CFR	IMR	MVD	Atherosclerosis	Epi- Spasm	Type of Spasm	Baseline Pd/Pa	FFR	CFR	IMR	MVD		
1	72	M	VSA	1	0	MVS	0.95	0.83	2	35	1	0	0	None	1.00	1.00	4.4	44	1	1	1
2	78	F	VSA	0	1	Focal	0.96	0.93	3.5	25	1	1	0	None	1.00	0.93	4.3	20	0	0	0
3	50	F	MVS	0	0	MVS	0.96	0.94	1.8	40.5	1	0	0	None	1.02	1.02	2.5	54.3	1	1	1
4	73	F	VSA	0	1	Focal	0.94	0.86	3.3	41.2	1	0	1	Focal	1.06	0.99	3.3	28.8	1	0	1
5	66	M	VSA	1	1	Diffuse	0.96	0.92	2.5	25.5	1	0	1	Diffuse	1.07	0.96	2.1	26.3	1	1	1
6	82	M	VSA	1	1	Focal	0.95	0.91	1.4	32	1	1	1	Focal	1.09	1.09	1.8	25.2	1	0	1
7	71	M	VSA	0	1	Diffuse	0.96	0.92	2.2	40.2	1	0	1	Focal	1.03	1.01	1.6	87.7	1	1	0
8	71	F	VSA	0	1	Focal	1	0.99	2	27	1	0	0	None	1.06	1.04	2.7	46.6	1	1	1
9	42	M	VSA	0	1	Focal	0.96	0.94	1.8	21.2	1	0	0	None	1.02	1.00	3.05	38.7	1	1	1
10	73	F	VSA	1	1	Diffuse	0.99	0.95	3	11.3	0	1	0	None	1.00	1.00	1.9	23.2	1	1	1
11	79	F	MVS	0	0	MVS	0.98	0.93	4	13.7	0	1	0	None	1.06	0.98	2	32	1	1	1
12	72	F	VSA	0	1	Focal	0.99	0.95	2.3	34.8	1	1	1	Focal	1.09	0.98	1.4	116.2	1	1	1
13	83	M	VSA	0	1	Focal	0.95	0.95	1.5	47.6	1	0	0	None	1.02	1.02	2.9	44.8	1	0	1
14	55	F	MVS	0	0	MVS	0.95	0.93	2.2	12.2	0	0	0	None	1.02	0.96	4.2	25.5	1	1	0
15	28	F	VSA	0	1	Diffuse	0.95	0.91	2.4	24.5	0	0	NA	NA	1.01	1.01	2.3	19	0	0	1
16	58	M	VSA	1	1	Diffuse	0.95	0.88	4.6	16.1	0	0	1	Diffuse	0.94	0.90	3.7	35	1	1	1
17	74	F	MVS	0	0	MVS	1.00	1.00	2.3	62.2	1	0	0	MVS	1.00	1.00	2.7	38.8	1	0	1
18	47	F	VSA	0	1	Focal	0.98	0.94	1.8	19.5	1	0	1	Diffuse	0.96	0.94	1.2	16.2	1	0	0
19	54	F	VSA	0	1	Diffuse	0.98	0.94	1.4	53.0	1	0	0	None	1.03	1.02	3.1	24.2	0	0	1
20	29	M	VSA	1	1	Diffuse	0.98	0.84	4.5	23.0	0	0	0	None	1.04	0.98	3.9	22.9	0	0	1
21	74	M	MVD	1	0	None	0.95	0.85	2.3	23.1	0	1	0	None	0.98	0.95	1.3	54.6	1	1	1
	71 (52, 74)	M/F 9/12	VSA/ MVS/ MVD 16/4/1	7 (33%)	15 (71%)		0.96 (0.95, 0.98)	0.93 (0.90, 0.95	2.3 (1.8, 3.2)	25.5 (20.4, 40.4)	14 (67%)	6 (29%)	7 (33%)		1.02 * (1.00, 1.05)	1.00 * (0.96, 1.02	2.7 (1.9, 3.5)	32.0 ^#^ (23.7, 45.7)	17 (81%)	12 (57%)	17 (81%)

Numbers were expressed as the numbers and values were expressed as the median with interquartile ranges. CFR, coronary flow reserve; FFR, fractional flow reserve; IMR, index of microcirculatory resistance; LAD, left anterior descending coronary artery; MVD, microvascular vasodilatory dysfunction; MVFT, microvascular vasodilatory function test; MVS, microvascular spasm; Pa, aortic pressure; Pd, distal pressure, RCA; right coronary artery. * *p* < 0.01 vs. the same values in the LAD, ^#^ *p* < 0.05 vs. the same value in the LAD.

## Data Availability

Date sharing is not applicable.
